# Supplementary material for: Genetic diversity and structure of baobab (Adansonia digitata L.) in southeastern Kenya
Source: R Soc Open Sci. 2019 Sep 11;6(9):190854. doi: 10.1098/rsos.190854 (PMC6774979; doi:10.1098/rsos.190854)
Supplement: Summary results from Structure assessed for the baobab populations in Kenya. [file rsos190854supp2.docx]

**Genetic diversity and structure of baobab (*Adansonia digitata* L.) in south-eastern Kenya**

Anna Chládová^1^, Marie Kalousová^1^, Bohumil Mandák^2,3^, Katja Kehlenbeck^4,5^, Kathleen Prinz^6^, Jan Šmíd^2^, Patrick Van Damme^1, 4, 7^, Bohdan Lojka^1*^

The affiliations and addresses of the authors:

^1^ Department of Crop Sciences and Agroforestry, Faculty of Tropical AgriSciences, Czech University of Life Sciences Prague, Kamýcká 129, CZ-165 00, Prague, Czech Republic

^2^ Department of Ecology, Faculty of Environmental Sciences, Czech University of Life Sciences Prague, Kamýcká 129, CZ-165 00, Prague, Czech Republic.

^3^ The Czech Academy of Sciences, Institute of Botany, Zámek 1, CZ-252 43 Průhonice, Czech Republic.

^4^ World Agroforestry Centre (ICRAF), PO Box 30677, 00100 Nairobi, Kenya

^5^ Rhine-Waal University of Applied Sciences, Marie-Curie-Straße 1, 47533 Kleve, Germany

^6^ Professorship for Systematic Botany, Friedrich-Schiller-University Jena, Philosophenweg 16, 07743 Jena, Germany

^7^ Department of Plants and Crops, Faculty of Bioscience Engineering, Ghent University, 9000 Ghent, Belgium

* Corresponding author: tel. +420 224 382 171, fax. +420 234 381 829; e-mail: [lojka@ftz.czu.cz](mailto:lojka@ftz.czu.cz)


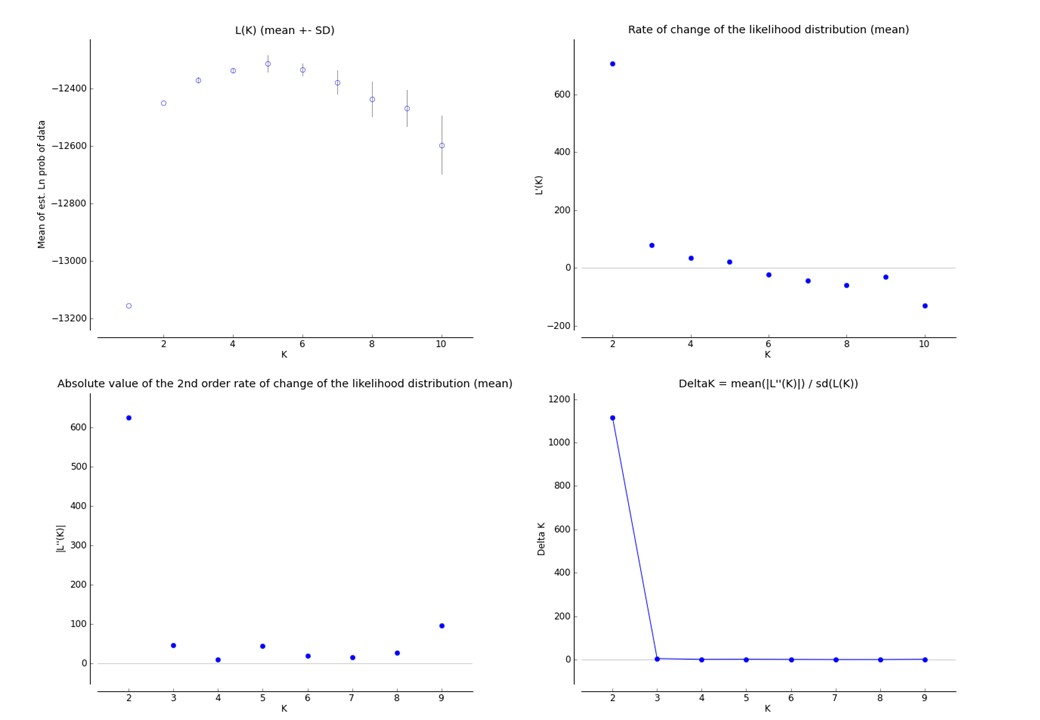


**Figure 1** Optimal number of genetic clusters in the studied *Adansonia digitata* seven populations in Kenya. The results obtained from STRUCTURE (Pritchard et al. 2000) were summarized in STRUCTURE HARVESTER (Earl & VonHoldt 2012).


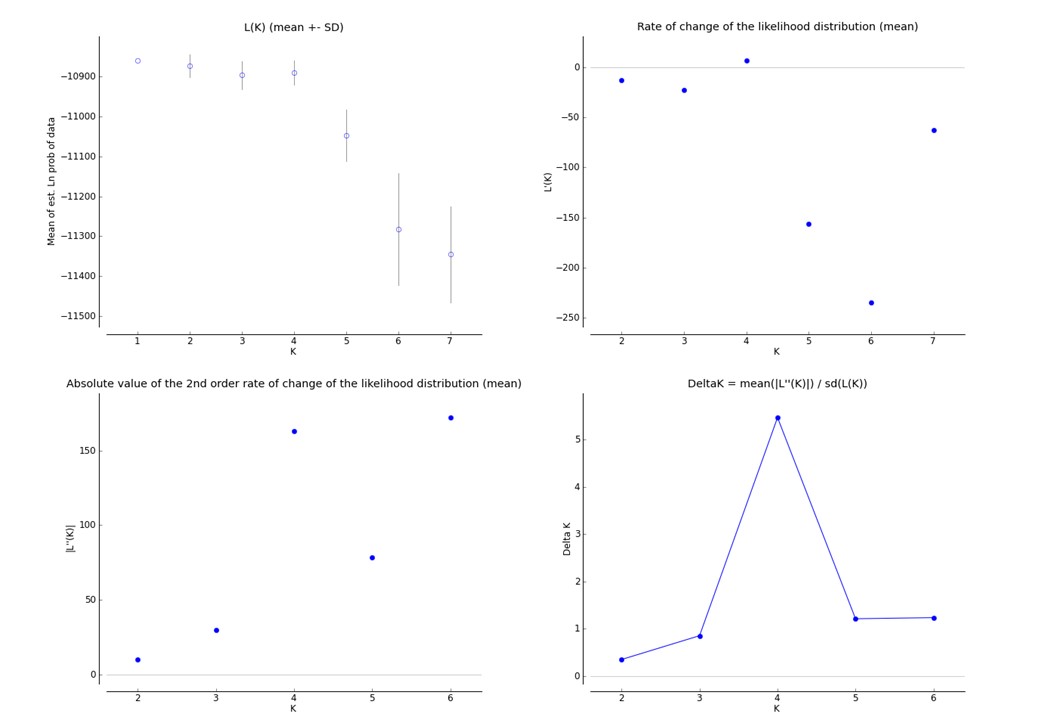


**Figure 2** Optimal number of genetic clusters in the studied *Adansonia digitata* inland populations in Kenya. The results obtained from STRUCTURE (Pritchard et al. 2000) were summarized in STRUCTURE HARVESTER (Earl & VonHoldt 2012).


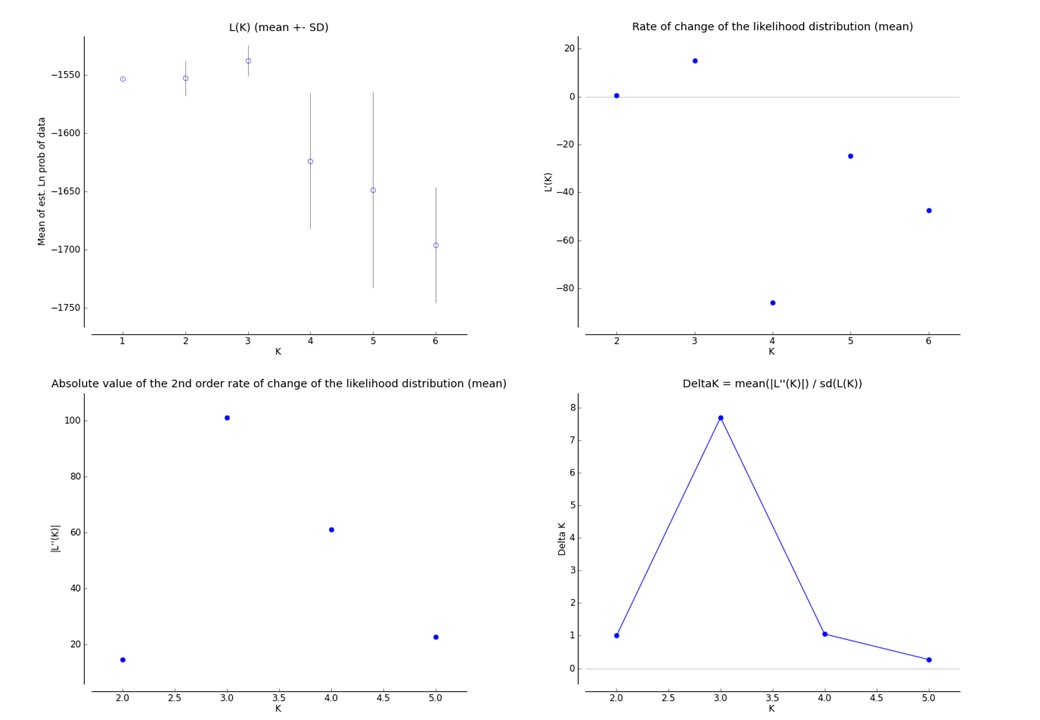


**Figure 3** Optimal number of genetic clusters in the studied *Adansonia digitata* coastal populations in Kenya. The results obtained from STRUCTURE (Pritchard et al. 2000) were summarized in STRUCTURE HARVESTER (Earl & VonHoldt 2012).

Pritchard JK, Stephens M, Donnelly P. 2000 Inference of population structure using multilocus genotype data. *Genetics* **155**, 945–959.

Earl DA, VonHoldt BM. 2012 STRUCTURE HARVESTER: A website and program for visualizing STRUCTURE output and implementing the Evanno method. *Conserv. Genet. Resour.* **4**, 359–361. (doi:10.1007/s12686-011-9548-7)
